# An Inulin-Specific Lectin with Anti-HIV-1 Reverse Transcriptase, Antiproliferative, and Mitogenic Activities from the Edible Mushroom* Agaricus bitorquis*

**DOI:** 10.1155/2019/1341370

**Published:** 2019-03-19

**Authors:** Guo-Qing Zhang, Qing-Jun Chen, Jing Hua, Zi-Lu Liu, Yue Sun, Xin Xu, Peng Han, He-Xiang Wang

**Affiliations:** ^1^Key Laboratory of Urban Agriculture (North China) of Ministry of Agriculture, College of Biological Science and Engineering, Beijing University of Agriculture, Beijing 102206, China; ^2^Beijing Key Laboratory for Agricultural Application and New Technique, College of Plant Science and Technology, Beijing University of Agriculture, Beijing 102206, China; ^3^State Key Laboratory for Agrobiotechnology and Department of Microbiology, China Agricultural University, Beijing 100193, China

## Abstract

A novel lectin (ABL) was purified from the dried fruiting bodies of* Agaricus bitorquis*. An efficient 3-step purification protocol involved two consecutive steps of ion exchange chromatography on Q-Sepharose and SP-Sepharose and gel filtration by FPLC on Superdex 75. ABL is a monomeric protein with the molecular mass of 27.6 kDa, which is different from other lectins from genus* Agaricus*. Its N-terminal amino acid sequence is EYTISIRVYQTNPKGFNRPV which is unique and sharing considerably high similarity of other mushroom lectins. The hemagglutinating activity of the lectin was inhibited by inulin. Based on hemagglutination tests, ABL prefers rabbit, human type A, and AB erythrocytes to human type B and O erythrocytes. The lectin inhibits the activity of HIV-1 reverse transcriptase and the proliferation of leukemia cell (L1210) with an IC_50_ value of 4.69 and 4.97 *μ*M, respectively. Furthermore, ABL demonstrates the highest mitogenic activity with a response of 24177.7 ± 940.6 [^3^H-methyl] thymidine counts per minute (CPM) at a concentration of 0.91 *μ*M.

## 1. Introduction

Lectins are carbohydrate-binding proteins of nonimmune origin that specifically recognize and bind to carbohydrate moieties of glycoproteins or glycolipids. They play crucial role in various biological and pathological processes, such as cellular signaling, cell-cell and host-pathogen interactions, differentiation, innate immune responses, serum glycoprotein turnover, tissue metastasis, etc. [1, 2]. Lectins are widely distributed in plants, fungi, animals, bacteria, and viruses. Among all the sources, plant lectins have been most extensively investigated including classification, structure, and functions. They act as the defensive system against animals and phytopathogenic microorganisms and also mediate the interactions of plants and their mycorrhizal fungi [3, 4]. Remarkably, numerous fungal lectins have been reported since their great therapeutic potentials as antitumor, antiproliferative, antivirus, immunomodulatory activities, etc. [5]. Mushrooms have been recognized for their nutritious and medicinal properties for thousands of years. More than 80% of reported fungal lectins come from mushroom species and play crucial roles in dormancy, growth, morphogenesis, morphological changes, and molecular recognition during their life cycles [1, 6]. Nowadays mushroom lectins have attracted considerable attention due to their treatment or prevention of cancer, viral diseases, atherosclerosis, hypertension, hypercholesterolemia, etc. [4, 7].

*Agaricus* is a genus of mushrooms from phylum Basidiomycota, class Agaricomycetes, order Agaricales, and family Agaricaceae with possibly over 300 members worldwide. Members of the genus* Agaricus* are characterized by having a fleshy cap and stipe and chocolate-brown spores. Some species are commercially manufactured including* A. bisporus*,* A. blazei*,* A. bitorquis*, etc. In recent years, lectins from* Agaricus* were widely reported including purification, properties, and therapeutic applications.* A. bisporus* lectins can inhibit the growth of colon cancer cell, breast cancer cell, and human retinal pigment epithelial cells [8].* A. blazei* lectin claimed to exert an in vitro antitumor activity in leukemic cells with no significant effects on normal lymphatic cells [9].

*A. bitorquis* is an edible mushroom species and can be commercially produced similar to the common button mushroom* A. bisporus*. It is an important supplement cultivation species of the button mushroom production in China. Up till now, only hemagglutination activity of* A. bitorquis* from Australia was reported [10]. In the present study, we aimed to purify a novel lectin from the artificially cultivated* A. bisporus* and find out its properties and potential therapeutic applications.

## 2. Materials and Methods

### 2.1. Assay for Hemagglutinating Activity

Hemagglutinating activity was determined in the 96-well microtiter plates with a final volume of 50 *μ*l. A serial twofold dilution of the lectin solution (25 *μ*l) was mixed with a 2 % rabbit erythrocyte suspension (25 *μ*l) in phosphate-buffered saline (pH 7.2) and incubated at room temperature for 1 h. The hemagglutination titer, one hemagglutination unit, was defined as the reciprocal of the highest dilution exhibiting hemagglutination. Specific activity is the number of hemagglutination units/mg protein [11]. The hemagglutination profiles of the lectin towards erythrocytes from other origins were similarly determined. Human blood (A, B, O, and AB types) was donated by healthy volunteers, and the blood type was confirmed by clinical tests.

### 2.2. Purification of Lectin

The mushroom* A. bitorquis* was artificially cultivated in woodland of Beijing mountain areas using a commercial strain from Hebei Province in China and followed the cultivation method of Chen et al. described [14]. Dried fruiting bodies (100 g) were homogenized and extracted in 0.15 M NaCl (1 L) at 4°C overnight. Subsequently, the homogenate was centrifuged at 9500 rpm for 20 min at 4°C. The supernatant was collected and ammonium sulphate was added to the supernatant to 80% saturation. The mixture was left at 4°C for 8 hours before another centrifugation at 9500 rpm for 20 min at 4°C. Then the precipitate was dissolved and dialyzed to remove ammonium sulphate before applying to a Q- Sepharose (GE Healthcare, USA) column (2.5×20 cm) which had previously been equilibrated with and was then eluted with 10 mM Tris-HCl buffer (pH 7.6). After removal of the unadsorbed fraction (Q1) containing slight hemagglutinating activity, two adsorbed fractions (Q2 and D3) were eluted with 200 mM NaCl and 1000 mM NaCl in the starting buffer, respectively. Fractions Q2 demonstrated strong hemagglutinating activity and were dialyzed for further purification on cation exchange chromatography of SP-Sepharose (GE Healthcare, USA) column (2.5×20 cm) with 10 mM NH_4_OAc buffer (pH 4.6). After removal of an unadsorbed fraction (SP1), two adsorbed fractions (SP2 and SP3) were eluted by using a linear concentration gradient of 0-1000 mM NaCl in the same buffer (pH 4.6). Lectin active fraction SP3 was finally applied to to gel filtration by fast protein liquid chromatography (FPLC, GE Healthcare, USA) on a Superdex 75 gel filtration column (0.2 M NH_4_HCO_3_ buffer, pH 9.4) using an AKTA Purifier (GE Healthcare, USA). The second fraction (SU2) was the purified lectin.

### 2.3. Determination of Molecular Mass

Molecular mass (*Mr*) of the purified lectin was determined based on FPLC-gel filtration and sodium dodecyl sulphate-polyacrylamide gel electrophoresis (SDS-PAGE). During the FPLC chromatography,* Mr* of natural proteins were calculated using the standard curve of Log* Mr* versus elution volume made by molecular mass standards (GE Healthcare, USA). SDS-PAGE was performed using the standard procedure with a 12% resolving gel and a 5% stacking gel.* Mr* of denatured proteins was obtained using another standard curve of Log* Mr* versus relative mobilities of molecular mass standards (Genview, USA).* Mr* of the present purified laccase was evaluated based on the two curves [13].

### 2.4. N-Terminal Amino Acid Sequencing

After SDS-PAGE, the purified enzyme on the gel was transferred to a polyvinylidene difluoride (PVDF, Bio-Rad, USA) membrane by electro-blotting and stained with CBB R-250. The stained band was then excised and analyzed by the automated Edman degradation method using an HP G1000A Edman degradation unit (Hewlett Packard Company, USA) and an HP1000 HPLC system (Hewlett Packard Company, USA) [11].

### 2.5. Assay of Hemagglutinating Inhibition by Carbohydrates

The hemagglutinating inhibition tests to investigate inhibition of lectin induced hemagglutination by various carbohydrates [11]. Serial twofold dilutions of sugar samples (200 mM to 1.56 mM) were prepared in phosphate-buffered saline. All of the dilutions were mixed with an equal volume (25 *μ*L) of the purified lectin solution with 16 hemagglutination units. The mixture was allowed to stand for 60 minutes at 4°C, and then mixed with 50 *μ*L of a 2% rabbit erythrocyte suspension. The assay for lectin activity was conducted as described above. The minimum concentration of the sugar in the final reaction mixture which completely inhibited 16 hemagglutination units of the lectin preparation was calculated. The carbohydrates tested included D-fructose, D-galactose, D-maltose, D-mannose, D-melibiose, D-xylose, L-rhamnose, L-sorbose, cellobiose, inulin, and raffinose.

### 2.6. Assay of HIV-1 Reverse Transcriptase (HIV-1 RT) Inhibitory Activity

Inhibitory activity towards human immunodeficiency virus type 1 (HIV-1) reverse transcriptase (RT) of the purified lectin was assayed using an enzyme-linked immunosorbent assay (ELISA) kit (Boehringer Mannheim, Germany) by determining the ability of HIV-1 RT to synthesize DNA [13]. A fixed amount (4-6 ng) of recombinant HIV-1 RT was used. The absorbance of the samples at 405 nm can be measured with an ELISA microtiter plate reader and was directly correlated to the level of RT activity. The inhibitory activity of the lectin was calculated as percentage inhibition compared with the control without any protein added. Lectin form the mushroom* H. erinaceum* was used as a positive control [15]. All treatments were performed in triplicate.

### 2.7. Assay of Antiproliferative Activity towards Tumor Cell Lines

Antiproliferative activity of the purified lectin was determined using the MTT (3-[4,5-dimethylthiazol-2-yl]-2,5-diphenyltetrazolium bromide) method towards the human liver cancer cell line Hep G2 and mouse lymphocytic leukemia cell line L1210 (American Tissue Culture Collection, USA) [11]. The cell lines (2×10^4^ cells/100 *μ*l) were seeded into 96-well culture plates in RPMI 1640 culture medium supplemented with 10% FBS, 100 mg/L streptomycin, and 100 IU/ml penicillin in a humidified atmosphere of 5% CO_2_ at 37°C for 24 h. Serials concentrations of the purified lectin (100 *μ*l per well) were added before further incubation for 48 h. Subsequently, the medium was removed and the plate was washed with phosphate-buffered saline (PBS). MTT solution (100 *μ*l per well) was then added, followed by further incubation for 1 h under the same conditions. Absorbance at 540 nm was measured after incubation with dimethyl sulfoxide (100 *μ*l per well) for 30 min. Reagent and control were included with the absence of cells or respectively. All treatments were performed in triplicate.

Viability (%) of tumor cells = [OD540 nm (sample)/ OD540 nm(control)] × 100.

### 2.8. Assay of Mitogenic Activity

In this assay, four BALB/C mice (20-25 g in body weight) were purchased from Beijing Vital River Experimental Animal Technical Co., Ltd. (China), and sacrificed by cervical dislocation. Spleens were removed aseptically and splenocytes were collected using a sterilized stainless steel sieve (100-mesh). The obtained splenocytes were subsequently resuspended to 5×10^6^ cells/ml in RPMI 1640 culture medium supplemented with 10% fetal bovine serum (FBS), 100 mg/L streptomycin, and 100 IU/ml penicillin. The splenocytes were seeded into a 96-well culture plate (Nunc, Denmark) with the final concentration of 7×10^5^ cells/100 *μ*l/well. Serials concentrations of the purified lectin (6.25-200 *μ*g/ml) in 100 *μ*l medium were added, followed by incubation at 37°C in a humidified atmosphere of 5% CO_2_ for 24 h. Each well was then pulsed with 10 *μ*l [methyl-^3^H]-thymidine (0.25 *μ*Ci, Amersham Biosciences, Sweden) and incubated for another 6 h under the same conditions. Finally, the splenocytes were harvested with an automated cell harvester onto a glass fiber filter. The radioactivity of each well was measured with a Beckman model LS 6000SC scintillation counter. Con A was used as a positive control because of its high potency [15]. All treatments were performed in triplicate.

## 3. Results

### 3.1. Lectin Purification

The lectin from fruiting bodies of the edible mushroom* A. bisporus* (abbreviated as ABL) was purified following an isolation protocol that entailed two consecutive steps of ion exchange chromatography of Q-Sepharose and SP-Sepharose, and a final gel filtration step of FPLC. The results of lectin purification at different steps were summarized in Table 1. The purification factor and specific activity of the purified lectin ABL was increased 4.3-, 12.1-, and 16.2-fold, respectively, after Q-Sepharose, SP-Sepharose, and FPLC. ABL possessed a hemagglutinating activity towards rabbit erythrocytes of 12064 U/mg and a 9.0% recovery of activity.

### 3.2. Properties of the Purified Lectin ABL

**Figure 1:**
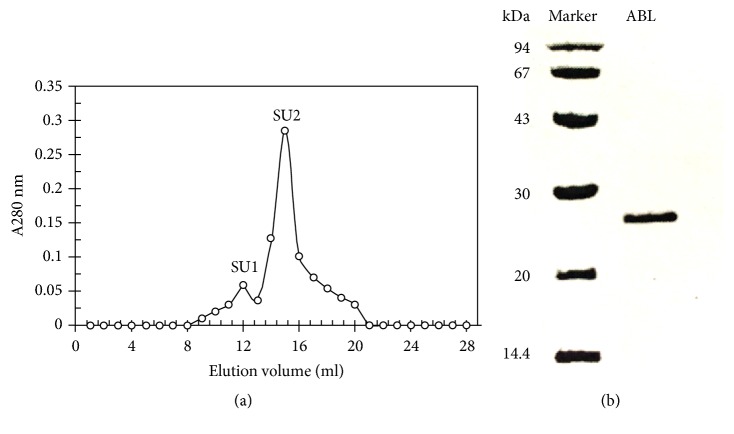
(a) FPLC-gel filtration on Superdex 75 HR 10/30 column. Eluent: 0.2 M NH_4_HCO_3_ buffer (pH 9.4). Fraction size: 0.8 ml. Flow rate: 0.5 ml/min. Fraction SU2 represents purified lectin ABL. (b) SDS-PAGE of ABL (right lane) and molecular weight markers (left lane). The markers were phosphorylase b (94kDa), bovine serum albumin (67kDa), ovalbumin (43kDa), carbonic anhydrase (30 kDa), soybean trypsin inhibitor (20kDa), and *α*-lactalbumin (14.4kDa), all from GE Healthcare.

**Figure 2:**
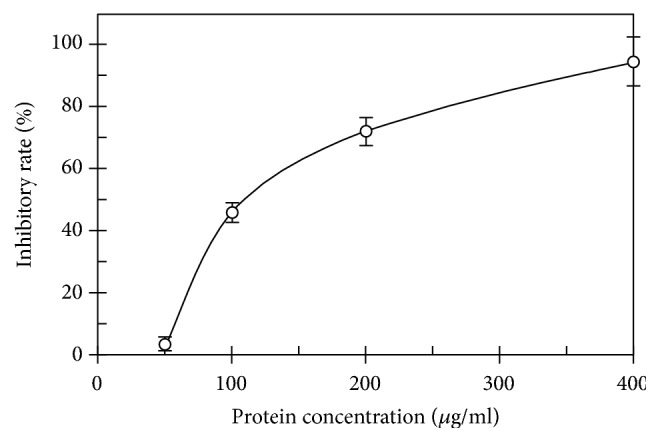
HIV-1 reverse transcriptase inhibitory activity of ABL (data represent means ± SD, n = 3).

**Figure 3:**
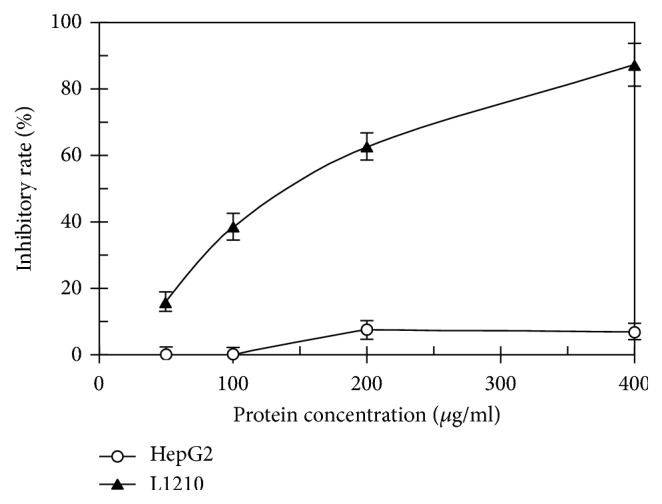
Inhibitory effect of ABL on proliferation of HepG2 and L1210 cancer cells. Cell proliferation was determined by MTT assay (data represent means ± SD, n = 3).

**Figure 4:**
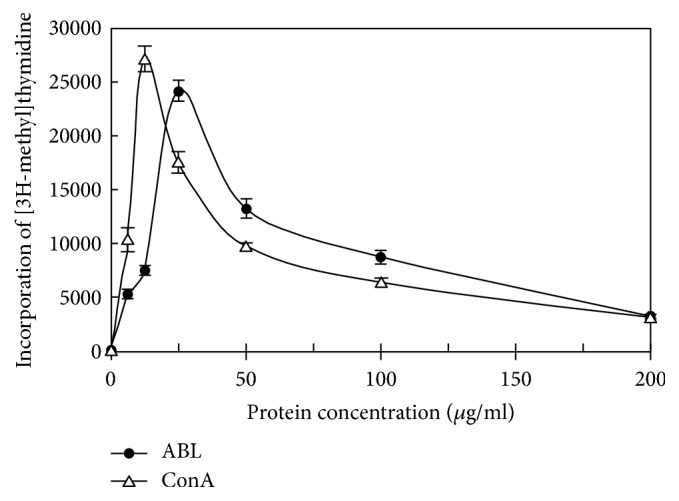
Mitogenic response of murine splenocytes induced by ABL and ConA (data represent means ± SD, n = 3).

ABL demonstrated a molecular mass (*Mr*) of 27.6 kDa when it was chromatographed on FPLC (Figure 1(a)) and exhibited one band with the same* Mr* in SDS-PAGE (Figure 1(b)). The first 20 N-terminal amino acids of ABL were sequenced to be EYTIS IRVYQ TNPKG FNRPV. A comparison of N-terminal amino acid sequence of ABL with that of other fungal lectins was shown in Table 2. Among the variety of sugars tested, inulin (with the assay concentration up to 6.25 mM) was able to inhibit the hemagglutinating activity of the purified lectin (Table 3). The purified lectin can agglutinate both rabbit and human erythrocytes with different capacity (Table 4). ABL preferred rabbit, human type A, and AB erythrocytes to human type B and O erythrocytes. ABL possessed significant HIV reverse transcriptase (RT) inhibitory activity with an IC_50_ value of 4.69 *μ*M (129.4 *μ*g/ml) (Figure 2) and inhibited the proliferation of mouse lymphocytic leukemia cells (L1210) with an IC_50_ value of 4.97 *μ*M (137.2 *μ*g/ml) (Figure 3). However, the inhibitory activity towards the proliferation of human HepG2 cells was very weak. Furthermore, ABL demonstrated the highest mitogenic activity with a response of 24177.7 ± 940.6 [^3^H-methyl] thymidine counts per minute (CPM) at a concentration of 0.91 *μ*M (25 *μ*g/ml), while Con A induced maximal mitogenic response with a response of 27144.0 ± 1193.8 CPM at a concentration of 0.12 *μ*M (12.5 *μ*g/ml) (Figure 4).

## 4. Discussion

Lectins are widely distributed among Basidiomycetes in not only fruiting bodies but also vegetative mycelia. About 61% reported lectins among Basidiomycetes come from order Agaricales, followed by Russulales and Boletales of 15% and 10%, respectively [16]. Up till now, lectins from genus* Agaricus* have been reported from* A. abruptibulbus*,* A. bisporus*,* A. arvensis*,* A. blazei*,* A. campestris*,* A. pilatianus*,* A. silvicola*, and* A. sylvaticus*, some of which were purified [4, 12, 17, 18]. Mushroom species from genus* Agaricus*, such as* A. bisporus* and* A. blazei*, are delicious and nutritious and possess health protection and clinical applications. Many of them can be artificially cultivated, among which* A. bisporus* is one of the most popular and widely cultivated all over the world. In the present study, the strain* A. bitorquis* was isolated from Hebei Province in China and artificially domesticated by local horticulturists. The fruiting bodies we used were cultivated in mountain areas of Beijing, China. It is more and more popular in China since it can be artificially cultured using the production equipment for* A. bisporus* or just in the wild. However, few reports on its lectins can be found.

Owing to differential binding strength on the Q-Sepharose and SP-Sepharose exchange columns, the lectin ABL could be purified from the crude protein solutions with an acceptable recovery (harvest) rate of 9.0% which is quite higher than that of other lectins from mushroom* Stropharia rugosoannulata* (7.9%) [11],* Hygrophorus russula* (1.2%) [13], etc. Many reported lectins can be adsorbed on both anion exchange chromatography as DEAE-Cellulose and Q-Sepharose and cation exchange chromatography as CM-Cellulose and SP-Sepharose. The less chromatography processes were used; the higher recovery rate would be obtained. A lectin from* A. blazei* was purified using chromatography of DEAE-Toyopearl and Sepharose 4B with the recovery rate of 9.2%-16% [18]. A lectin from a wild toxic mushroom* Inocybe umbrinella*, which is from the same order as* A. bitorquis*, was purified with a recovery rate of 11% following a protocol comprised ion exchange chromatography on DEAE-Cellulose, and CM-Cellulose, and gel filtration on Superdex 75 [19]. However,* H. russula* lectin was isolated with a very low recovery rate of 1.2% and a long purification procedure encompassing five successive ion exchange chromatography on CM-Cellulose, DEAE-Cellulose, Q-Sepharose, SP-Sepharose and Mono S [13]. Therefore, simple and efficient purification processes are the important guarantees for protein purification and the recovery rate.

Based on both FPCL and SDS-PAGE, the present lectin was determined to be a monomeric protein with a molecular mass of 27.6 kDa which is different from many other lectins reported. Since the first lectin was reported from a famous toxic mushroom* Amanita muscaria* (Fly agaric) in the year 1910, more than 300 mushroom lectins have been reported which demonstrate different molecular masses and subunit structure [4, 16]. The common mushroom* A. bisporus*, which is very close to* A. bitorquis* genetically, was reported to have between two and four hemagglutinin proteins of 58 kDa to 64 kDa, composed of identical subunits of 16 kDa [20]. Molecular mass of the lectin from* A. blazei* was determined to be 60-70 kDa using gel filtration and SDS-PAGE with four identical subunits of ~16 kDa [18]. A thermostable 30.4-kDa lectin from dried fruiting bodies of* Agaricus arvensis* was reported to be a dimer made up of two 15.2 kDa homogeneous subunits [12]. Lectins from* Polyporus squamosus* (28 kDa) and* Xylaria hypoxylon* (28.8 kDa) possess a very close molecular mass with ABL, but both of them are homodimeric lectins. To date, a lectin from* Pholiota squarrosa* possesses the lowest molecular mass of 4.5 kDa [4].

Although ABL demonstrates a unique molecular mass and subunit structure comparing with other lectins from the genus* Agaricus*, N-terminal sequence of ABL shares considerably high homology with that of* A. bisporus* (85%, AAA85813.1) and* A. bisporus* var.* bisporus* H97 (70%, XP_006455555.1), but low homology with that of* A. arvensis* (30%) (Table 2) [12]. It also shows high sequence similarity of about 55-65% with fungal lectins from other genera, such as* Pleurotus cornucopiae* (65%, BAB63923.1),* Sistotremastrum niveocremeum* (65%, KZS90447.1),* Boletopsis grisea* (60%, ANW37921.1),* Sclerotium rolfsii* (60%, 2OFC_A),* Metarhizium anisopliae* (55%, KFG85697.1), and* Punctularia strigosozonata* (55%, XP_007383986.1). On the other hand, less than 10% homology of N-terminal amino acid sequence has been found between ABL and lectins from* H. russula* (10%) [13] and* Stropharia rugosoannulata* (5%) [11].

The hemagglutinating activity of the purified lectin cannot be inhibited by a variety of monosaccharides tested, but can be inhibited by the polysaccharides inulin which is a heterogeneous collection of fructose polymers. Many mushroom lectins can be inhibited by monosaccharides or their derivatives. The hemagglutinating activity of* X. hypoxylon *lectin is inhibited by xylose [4]. The hemagglutinating activity of the lectin from* I. umbrinella* is inhibited by both D-galactose and D-melibiose [19]. Lectin from* A. bisporus* is inhibited by N-acetyl-D-galactosamine, while lectins from other* Agaricus* species, including* A. blazei*,* A. campestris*, and* A. edulis*, are devoid of inhibitory activity by common monosaccharides [4]. It suggests that the present lectin from artificially cultivated* A. bitorquis *manifests a distinctive carbohydrate-binding specificity with other* Agaricus* lectins as indicated by the ability of only inulin to inhibit its hemagglutinating activity. In addition, inulin can also inhibits the hemagglutinating activity of other lectins from Agaricomycetes, as* Pholiota adiposa*, and* R. lepida* [4].

Most lectins are nonspecific and can agglutinate erythrocytes of all human blood groups without any noticeable specificity [16]. The present lectin ABL can agglutinate human type A, B, O, AB, and rabbit erythrocytes, while hemagglutination in human type A, AB, and rabbit erythrocytes will be stronger than that in human type B and O erythrocytes. Just like ABL, lectins from* A. bisporus* and* A. campestris* also manifest hemagglutinating activity towards human type A, B, O, AB, and rabbit erythrocytes.* A. abruptibulbus* lectin possesses agglutination activity towards human type O erythrocytes double than human A, B, and rabbit erythrocytes [16]. Moreover, a homodimeric lectin from* Clitocybe nebularis* agglutinates human type A erythrocytes with highest affinity, followed by human type B, O, and bovine erythrocytes [21].

ABL demonstrate potential therapeutic applications with considerably high anti-HIV, antitumor, and mitogenic activities. The lectin inhibits the HIV-1 RT activity with an IC_50_ value of 4.69 *μ*M, which is more efficient than a lectin from* A. bisporus* with an IC_50_ value of 8 *μ*M [16].* I. umbrinella* lectin exhibits HIV-1 RT inhibitory activity with an IC_50_ value of 4.7 *μ*M, which is just the same as ABL [19]. Other mushroom lectins show extremely high inhibitory activity towards HIV-1 RT with very low IC_50_ values, such as lectins from* P. adiposa* (IC_50_ value of 1.9 *μ*M) [22]. Moreover, lectins from* S. rugosoannulata* and* Hericium erinaceus* inhibit HIV-1 RT with a higher IC_50_ value of 10 and 31.7 *μ*M, respectively [11, 15]. On the other hand, many mushroom lectins do not exhibit HIV-1 RT inhibitory activity.

ABL displays inhibitory activity against mouse lymphocytic leukemia cells L1210 with an IC_50_ value of 4.97 *μ*M, but has no significant effect on human HepG2 cells. Many lectins isolated from order Agaricales exhibit significant inhibitory activity against tumor cells.* A. bisporus* lectin (50 mg/ml) can inhibit the proliferation in human colon cancer HT29 cells without cytotoxicity [16]. Lectin from the mushroom* Armillaria luteo-virens* inhibit MBL2, HeLa, and L1210 cells with an IC_50_ value of 2.5, 5, and 10 *μ*M, respectively. However, proliferation of HepG2 cells was not affected, which is similar with that of ABL in the present study [23]. It is worth mentioning that the lectin from* Armillaria luteo-virens* was also inulin-specific just like the present one. While another inulin-specific lectin from* P. adiposa* demonstrated antiproliferative activity towards both Hep G2 and MCF7 cells with an IC_50_ value of 2.1 and 3.2 *μ*M, respectively [22]. That is to say, the sugar specificity is very important factor for antiproliferative activity, but not the only one.

Mushroom lectins share great diversity in different species and even one species in different growth conditions. That is why the lectins are unique in the sugar specificity and antiproliferative potential. Tumor cell surfaces vary in composition of glycoconjugates and their terminal saccharide units, which is believed to be correlated to tumor metastasis [4]. Lectins may display antiproliferative potential by cross-linking the glycoconjugates of tumor cell surfaces or through immunomodulatory effects [7]. Lectin from* Tricholoma mongolicum* inhibits mouse mastocytoma cells* in vitro* and Sarcoma S 180 cells in Bal b/c mice by modulation of the immune system rather than direct cytotoxicity [24]. Lectin and fermentation extract from European mistletoe (*Viscum album*) have been investigated in clinical treatments of malignant melanoma, which is shown to be safe and without any further tumor enhancement [25]. The potent antiproliferative activity of ABL is remarkable and hopefully it can be developed into an agent for cancer therapy, and also diagnostic and experimental tools to study the various features of cell growth and differentiation.

Many mushroom lectins possess the remarkable property of stimulating the transformation of lymphocytes from small resting cells to large blast-like cells that may undergo mitosis, such as* A. luteo-virens* [23],* Agrocybe cylindracea* [26], and* B. edulis* [27]. The present lectin is capable of eliciting a mitogenic response from mouse splenocytes with a response of 24177.7 CPM at a concentration of 0.91 *μ*M (25 *μ*g/ml), which is similar to that of* A. luteo-virens* lectin of 25000 CPM at a concentration of 1 *μ*M [23]. Although the magnitude of the maximal mitogenic response to ABL (24177.7 CPM of 0.91 *μ*M ) is less than that to Con A (27144.0 CPM of 0.12 *μ*M), it manifests higher mitogenic activity than lectins from* A. cylindracea* (6000 CPM of 2 *μ*M) [26] and* B. edulis* (14000 CPM of 1 *μ*M) [27]. Moreover,* S. commune* lectin exhibits a mitogenic response of 23000 CMP at lectin concentration of 4 *μ*M [28].

## 5. Conclusion

In summary, a novel lectin was purified for the first time from the artificially cultivated mushroom* A. bitorquis*. It possesses unique properties of different molecular mass and single subunit structure comparing with other lectins from the genus* Agaricus*. It is an inulin-specific lectin and prefers rabbit, human type A, and AB erythrocytes. It manifests significant HIV-1 RT inhibitory activity, antiproliferative activity towards cancer cells, and mitogenic activity towards murine splenocytes. In this regard it is noteworthy that the present lectin demonstrates potential therapeutic applications.

## Figures and Tables

**Table 1:** Yields and hemagglutinating activities of various chromatographic fractions derived from 100g dried fruiting bodies.

Purification step	Yield (mg)	Total activity (U)	Specific activity (U/mg)	Recovery of activity (%)	Purification fold
Crude extract	5230	3.9 × 10^6^	745	100.0	1.0
Q-Sepharose (Q2)	814	2.6 × 10^6^	3218	67.2	4.3
SP-Sepharose (SP3)	57	5.1 × 10^5^	9026	13.2	12.1
FPLC (SU2)	29	3.5 × 10^5^	12064	9.0	16.2

**Table 2:** Comparison of the N-terminal sequence of ABL with other fungal lectins.

Species	N-terminal sequence	Max ident	Accession number / Ref.
*Agaricus bitorquis*	1 **EYTIS** **IRVYQ** **TNPKG** **FNRPV** 20	100%	Present study
*Agaricus bisporus*	2 T**YTIS** **IRVYQ** **T**T**PKG** **F**F**RPV** 21	85%	AAA85813.1
*Agaricus bisporus *var.* bisporus* H97	2 S**YTI**T **IRVYQ** **T**D**P**NA **F**F**RRV** 21	70%	XP_006455555.1
*Agaricus arvensis*	1 T**Y**AVL NF**VY**G 10	30%	[12]
*Pleurotus cornucopiae*	2 S**YTI**K V**RVYQ** **TNP**NA **F**F**R**I**V** 21	65%	BAB63923.1
*Sistotremastrum niveocremeum*	2 A**YTI**T **I**H**VYQ** **TNPK**I **F**FKI**V** 21	65%	KZS90447.1
*Boletopsis grisea*	2 S**Y**K**I**C V**RVYQ** **TNP**NA **F**F**R**I**V** 21	60%	ANW37921.1
*Sclerotium rolfsii*	2 T**Y**K**I**T V**RVYQ** **TNP**NA **F**FH**PV** 21	60%	2OFC_A
*Metarhizium anisopliae*	2 S**YTI**T VQ**VYQ** **TNP**NA **F**FHL**V** 21	55%	KFG85697.1
*Punctularia strigosozonata*	2 S**YTI**T VS**VYQ** **TNP**NC **F**FNI**V** 21	55%	XP_007383986.1
*Hygrophorus russula*	1 TIGNA KP**V**LV QQEIV GG**R**RI 20	10%	[13]
*Stropharia rugosoannulata*	1 IKSGV Y**R**IVS WQGAL GPEAR 20	5%	[11]

Amino acid residues identical to corresponding residues of ABL are underlined.

**Table 3:** Effect of various carbohydrates on hemagglutination induced by ABL (16 hemagglutinating units).

Sugar (mM)	100	50	25	12.5	6.25	3.125	1.56	0.78
D-Fructose	+	+	+	+	+	+	+	+
D-Galactose	+	+	+	+	+	+	+	+
D-Maltose	+	+	+	+	+	+	+	+
D-Mannose	+	+	+	+	+	+	+	+
D-Melibiose	+	+	+	+	+	+	+	+
D-Xylose	+	+	+	+	+	+	+	+
L-Rhamnose	+	+	+	+	+	+	+	+
L-Sorbose	+	+	+	+	+	+	+	+
N-acetyl-D-galactosamine	+	+	+	+	+	+	+	+
Cellobiose	+	+	+	+	+	+	+	+
Inulin	−	−	−	−	−	+	+	+
Raffinose	+	+	+	+	+	+	+	+

+, hemagglutination activity; −, no hemagglutination activity; the experiment was repeated twice and the results were reproducible.

**Table 4:** Specific hemagglutinating activity of ABL towards erythrocytes from different origins.

Species	Strain/line	Hemagglutinating activity (U)
Human		
A	Chinese	16
B	Chinese	12
O	Chinese	8
AB	Chinese	16
Rabbit	Cuniculus	16

The experiment was repeated twice and results were reproducible.

## Data Availability

The data used to support the findings of this study are available from the corresponding author upon request.
